# Supplementary figures and images for: Asymptomatic neonatal herpes simplex virus infection in mice leads to persistent CNS infection and long-term cognitive impairment
Source: PLoS Pathog. 2025 Feb 7;21(2):e1012935. doi: 10.1371/journal.ppat.1012935 (PMC11828378; doi:10.1371/journal.ppat.1012935)

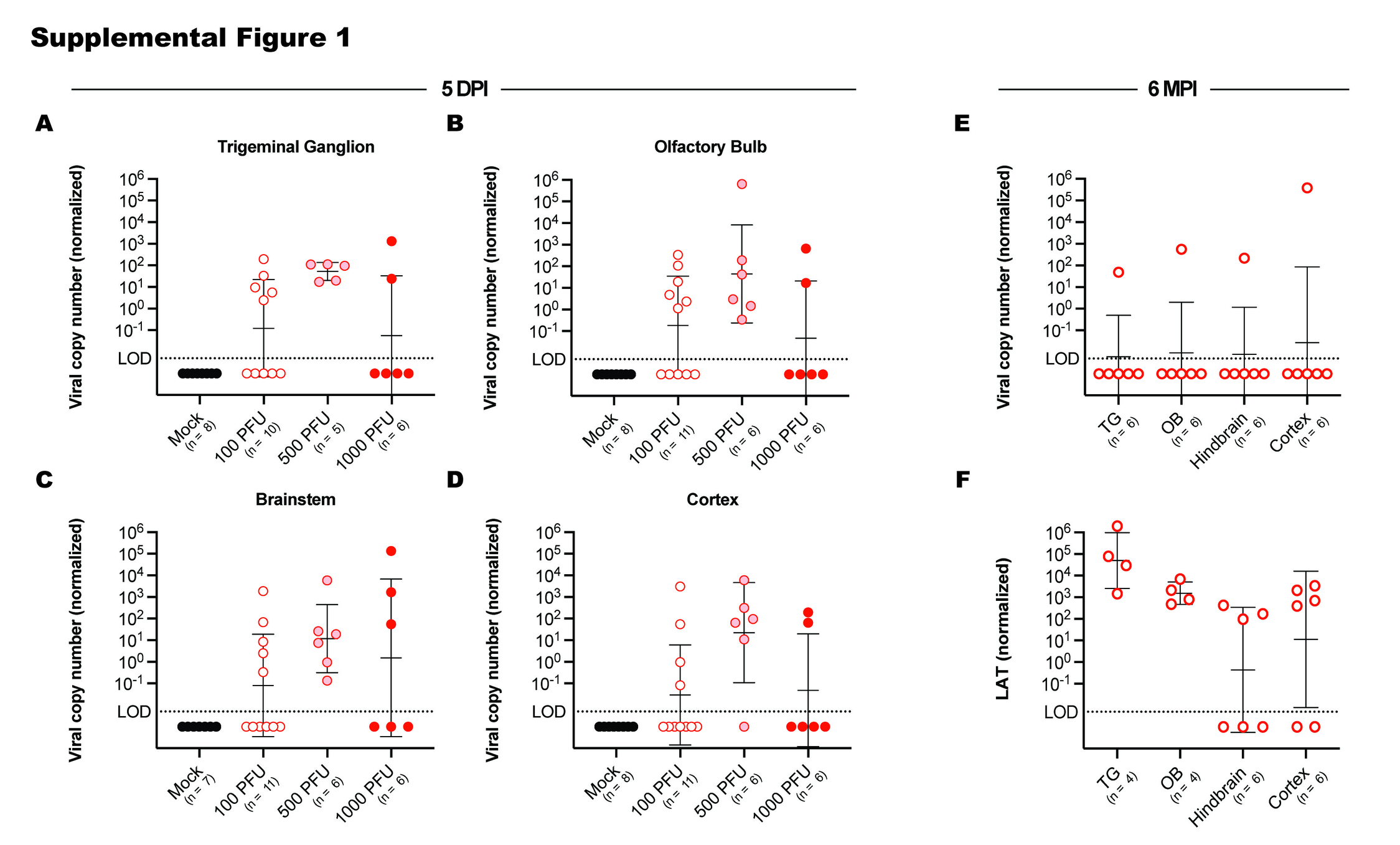

Supplement: S1 Fig — HSV genome copy number in the trigeminal ganglia (A), olfactory bulbs (B), cerebral cortex (C), and hindbrain (D) of 100 PFU nHSV-1 infected mice 5 dpi, relative to mouse single-copy adipsin gene, as determined by RT-PCR. (E) HSV genome copy number in the trigeminal ganglia, olfactory bulbs (OB), hindbrain, and cerebral cortex of 100 PFU nHSV-1-infected mice at 6 mpi. (F) Quantification of HSV-1 latency-associated transcript (LAT) in the trigeminal ganglia, olfactory bulbs (OB), hindbrain, and cortex of 100 PFU nHSV-infected mice at 6 mpi. Error bars represent standard deviation from the geometric mean. (TIF) [file ppat.1012935.s001.tif]

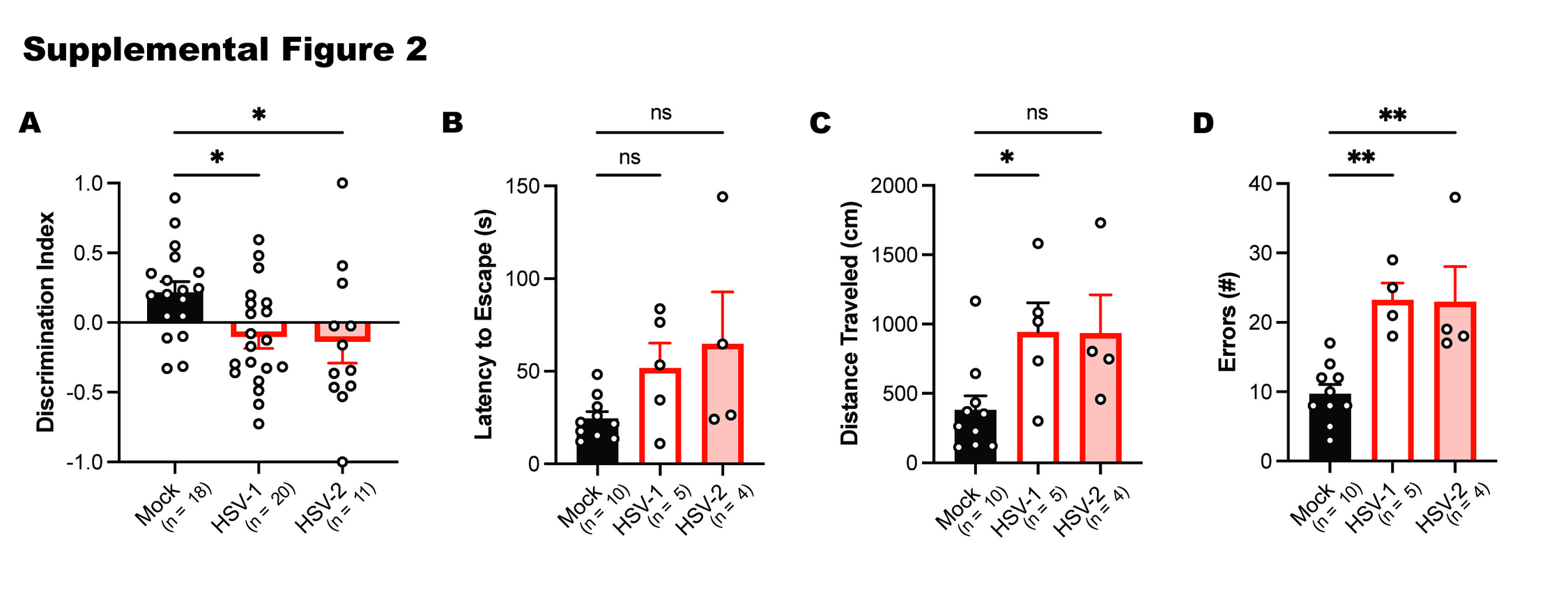

Supplement: S2 Fig — (A) Performance of mock-infected (n = 18), strain 17 (st17) nHSV-1-infected (n = 20), and strain 333 nHSV-2-infected mice (n = 12) on the novel object recognition (NOR) task at 5 mpi. Bar graph shows the mean discrimination index for the novel object. Discrimination index = (Timenovel − Timefamiliar)/Timetotal. (B-D) Performance of mock-infected (n = 10), strain 17 (st17) nHSV-1-infected (n = 5), and strain 333 nHSV-2-infected mice (n = 4) on the MBM task at 5 mpi. Mean latency to escape on the first trial of reversal testing in the modified barnes maze (MBM). (E) Mean distance traveled in centimeters (cm) during the first trial of reversal testing in the MBM. (F) Mean errors per trial committed during the first trial of reversal testing in the MBM. Statistical significance determined by one-way ANOVA with Tukey’s and Dunnett’s multiple comparisons post-hoc tests (D-E). *p < 0.05, **p < 0.01. Error bars represent standard error (SEM). (TIF) [file ppat.1012935.s002.tif]

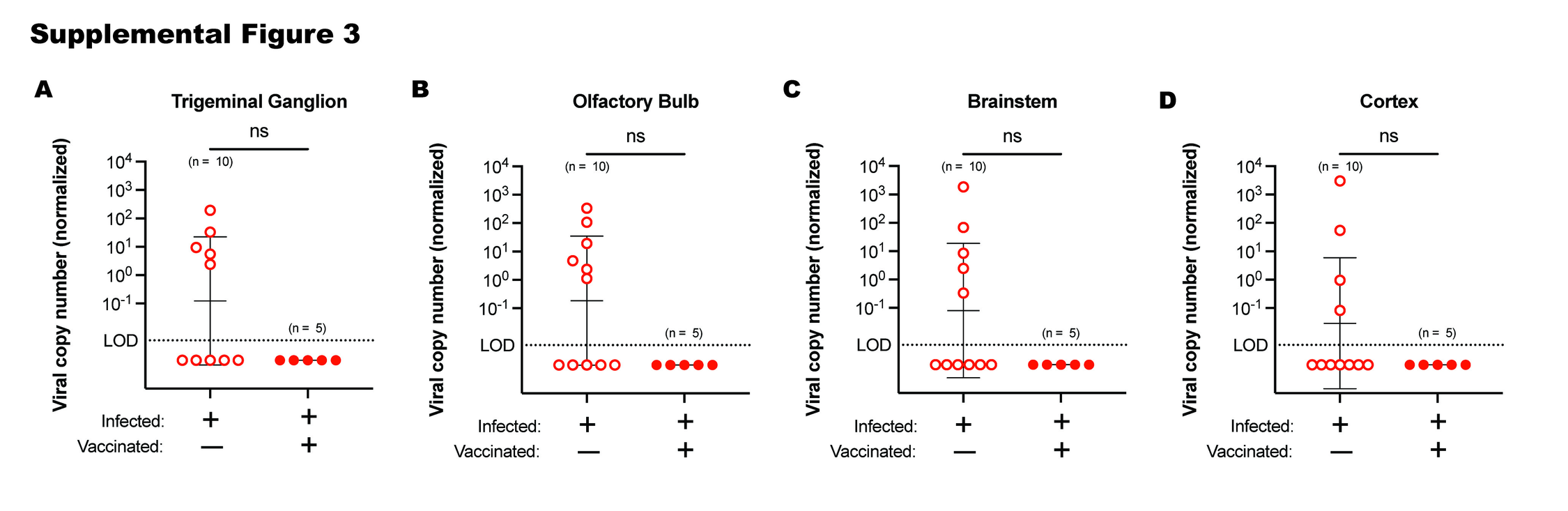

Supplement: S3 Fig — HSV genome copy number in the trigeminal ganglia (A), olfactory bulbs (B), cerebral cortex (C), and hindbrain (D) of 100 PFU nHSV-1 infected pups of dl5-29-vaccinated dams 5 dpi, relative to mouse single-copy adipsin gene, as determined by RT-PCR. Statistical significance was determined by unpaired t-test. ns = not significant. Error bars represent standard deviation from the geometric mean. (TIF) [file ppat.1012935.s003.tif]
