# Supplementary material for: Asymptomatic neonatal herpes simplex virus infection in mice leads to persistent CNS infection and long-term cognitive impairment
Source: PLoS Pathog. 2025 Feb 7;21(2):e1012935. doi: 10.1371/journal.ppat.1012935 (PMC11828378; doi:10.1371/journal.ppat.1012935)
Supplement: Attachment — Submitted filename: Plos Path Dutton et al response to reviewers.pdf [file ppat.1012935.s004.pdf]

Dartmouth
GEISEL SCHOOL OF MEDICINE
DEPARTMENT OF MICROBIOLOGY AND IMMUNOLOGY

January 17th, 2025.

Re: "**Asymptomatic neonatal herpes simplex virus infection in mice leads to long-term cognitive impairment**." PPATHOGENS-D-24-02176

Dear PLoS Pathogens Editorial Board Members,

We sincerely thank the reviewers whose comments have helped strengthen this study. We have made many rewordings/clarifications and performed a number of new experiments since the first submission that collectively answer questions raised by the reviews. The most salient set of experiments, included now as panels in Figure 1 and Figure S1, demonstrates the persistence of HSV genomes and latency-associated transcripts (LATs) in the CNS over the 6-month time frame of our model. This finding supports the notion that HSV persists in the mouse CNS for at least 6 months following neonatal infection with only 100 PFU HSV-1 in a subset of infected mice. We have therefore changed the title to reflect this, it now reads "Asymptomatic neonatal herpes simplex virus infection in mice leads to *persistent CNS infection and* long-term cognitive impairment."

This letter outlines our point-by-point responses to the comments of the reviewers, and describes additional data added to the revised manuscript.

Part II – Major Issues: Key Experiments Required for Acceptance
Responses to reviewer #1:

1. Immunofluorescence:

a. "*Please perform image analysis to quantify the gC fluorescence per image ……If possible ….quantified per brain region, e.g. brainstem, midbrain, cortex.*"

- We have now performed this analysis and it is described on lines 192-194 and shown in Figure 1E.

b. "*As a companion to Fig. 1C, include both an image and quantification for the lack of gC detection in 100 PFU infected brains. This is to support the text claim at line 193-194.*

- The sole intention of Fig 1C (now 1D) is to demonstrate the potential routes of spread and localization in the CNS of HSV-derived protein following low-dose intranasal infection. This has not been previously shown. No protein was visible at 100pfu by IF, so we used 1000pfu as a surrogate to show where the virus can spread. There is therefore no reason to show the negative 100pfu images since no data can be gleaned from them.

c. "*The authors stained for gC which would only be detected at late stages during productive infection.. This does not rule out abortive lytic infection vs. latency. In an ideal world the authors would also stain for IE or E viral proteins (e.g. ICP4, ICP8) ….we would ask the authors to clarify in the text what the presence of gC means in the context of infection and give those respective caveats.*"

- The presence of a true-late antigen (gC) without expression of the IE and E genes would be unprecedented. Hence, looking for gC rather than IE or E genes supports the idea of lytic, rather than abortive infection at this early time point. We have now demonstrated the presence of persistent DNA genomes and LATs indicating latency at 6 months post infection. We have clarified and provided caveats in the text (lines 208-213).

2. Characterization of HSV1 CNS infection after maternal immunization.

a. *"The claim at line 349-352 is overstating the data presented "Given that virus was undetectable in brains of offspring from vaccinated dams at 5 dpi, the development of neurologic morbidity is likely dependent on HSV establishing a threshold of acute and latent neonatal CNS infection*."

- We have removed this text.

b. "*You cannot state "virus was undetectable in brains" (line 349-350) when you only show data for HSV1 DNA in the midbrain. Please include data for other regions similar to what you show in Sup. Fig. 1 (TG, olfactory bulb, cortex, hindbrain).*"

- We have incorporated these data into new supplemental figures (Figs S1 and S3).

c. *"With the current data presented, it's unclear whether the virus fails to establish latency after maternal immunization or if it does not reach the CNS during primary infection. Please comment.*"

- We have commented on this and added data (line 360-364 and Fig S3).

3. Low PFU model=asymptomatic.

a. "*We would ask the authors to include additional metrics for the mice to support the model as asymptomatic*."

- We have added a graph to indicate weight gain over time for infected/control mice (Fig. 1B).

Responses to Reviewer #2

1. "*The authors should perform assays to determine …..e.g., histology, quantification of proteins involved in inflammation, expression of appropriate neuronal and glial cell markers, neuronal death, etc). In case such changes are not observed, how do the authors explain the long-term consequences of infection?*"

- We have performed such assays without seeing visible changes. It is likely that relatively small and undetectable changes in physiology, synaptic connectivity, and function etc during the neonatal period can lead to permanent cognitive changes. Based on our new data showing persistent viral DNA (Fig S1) we have proposed explanations/hypotheses for future studies (several areas, but see esp. lines 425-432).

2. "*Are there any anatomical and/or physiological changes associated with dementia in the CNS of mice with cognitive impairment?"*

- As above, we were unable to detect any obvious changes, although small changes in anatomy and physiology can lead to significant cognitive changes. We comment on this in the manuscript (lines 425-432).

Responses to Reviewer #3

"…..there is no assessment of the infection status of the adult mice subjected to cognitive tests. In this model, do the mice clear the infection or do they have a latent (or persistent) infection?"

- As stated above, we have addressed this experimentally (Fig S1 and S3) showing clear evidence of long-term persistent infection, as now reflected in a new amended title for the study.

Part III – Minor Issues: Editorial and Data Presentation Modifications

Reviewer #1:

1. *"Please remove the text at lines 387-389 "The small dose of HSV inoculum needed to induce memory impairment suggests that viral-induced cognitive decline may not depend on reactivation*

events in our model." While the model did not include or test any intentional reactivation triggers, the authors cannot assume spontaneous reactivation isn't occurring in their model & contributing to the cognitive changes detected."

- We agree, and the text has been altered (line 427-429) since the positive DNA PCR at 6 months implies that spontaneous reactivation could be occurring.

2. "*The authors fail to comment on why a difference between mock and HSV-1 infection were only seen at day 1 via the MBM test. What would this result indicate regarding the actual cognitive delay or type of damage? Please include a discussion of this"*.

- The behavior observed is consistent with increased perseveration and impaired behavioral flexibility. We have added a discussion of this point (line 304-306).

3. Data presentation:

a. "All bar graphs (Fig. 1D, Fig. 2 bottom panels, Fig. 3 D-G, Fig. 4D-F, Fig. 5C-E, Sup Fig. 1A-D, Sup Fig. 2 A-D) need to be updated to show individual data points for each mouse/sample included."
b. "Include "ns" labels for statistical tests performed that are not significant (Fig 3G, 4B-C, Sup Fig. 2B)"

- This has been amended throughout all relevant figures.

Reviewer #2:

1. "*Is the text referring to the statistics employed in Figure 1A correct? Or does it refer to panel B*?"
   - It refers to panel B and this has been amended.

2. "*It will be more informative to show the performance of each individual mouse in the tests with a symbol, rather than using bars to represent the mean/average"*.
   - This has been amended.

3. "*It is not clear if the same mice undergo each individual test or whether different mice were employed in the different tests. Please, clarify this. If the same mice were used in different tests, do the same individual mouse always perform worse/better in the test? Or are there mice that perform well in some tests and bad in others*?"
   - This has been clarified – different mouse cohorts are used for each test (line 566-568).

4. "*Was viral DNA detected in tissues/organs other than CNS in the offsprings of vaccinated dams following challenge with HSV*?"
   - We did not test for this.

5. "*Line 418: Typo in "Additinally"*
   - This has been amended to read "additionally".

6. *Line 419: "antibody prevents" should be "antibodies prevent*".
   - This has been amended.

7. "*Line 483: Dr. David Knipe is a co-author of this manuscript so I suppose it is not necessary to indicate that he provided the replication-deficient virus. Similarly, he should not be included in the Acknowledgements"*.
   - This has been amended.

8. "*Line 602 and others: Separate the cipher from the unit of measure (i.e., 10s --- 10 s*)".
   - This has been amended.

9. *Supplemental Figure 1: Include trigeminal ganglion in the title of the figure, this is not a component of the brain*.
• This has been amended.

Reviewer #3:

1. …"*The authors justify their use of the low-dose neonatal infection model by noting that asymptomatic shedding is common for genital herpes……..Altogether, it is not necessary to use asymptomatic shedding as a justification for this mouse model or study (the model is fine and it is reasonable to investigate outcomes of subclinical neonatal HSV infections)".*
• It is likely that maternal asymptomatic shedding would result in a low level of virus being transmitted to the neonate. This model is therefore justified from both the perspective of maternal asymptomatic shedding, as well as neonatal asymptomatic infection.

2. *"In addition to noting prior work assessing cognitive and behavioral outcomes in mice after HSV infection, the authors may consider discussing studies with other viruses, particularly with neonatal and congenital infection models. Examples of references to consider………..".*
• Additional references have been added.

3. "*Throughout: change "symptoms" to "clinical signs", "disease signs", or similar when referring to mice*".
• This has been amended.

4.*" Throughout: replace bar charts with scatter plots to better convey data distribution*".
• This has been amended throughout all of the figures that contain bar charts.

5."*Consider moving HSV-2 data into the main figure with HSV-1 (supplementary Fig 2*)."
• Due to slight differences in experimental design it is more appropriate to keep these datasets separate.

6. *"Line 169-170: consider deleting "in this era of vaccine hesitancy" (not necessary)"*.
• With vaccine confidence on the wane, it is important to emphasize vaccine safety in this context.

7. "Li*ne 197: trigeminal ganglia are not part of the brain".*
• This has been amended.

8. "*Line 423-424: delete this sentence (not necessary)".*
• Again, we believe it is important to emphasize this novel aspect of vaccine safety.

9. *Line 427-430: Consider tempering this claim. The present study concludes that viral infection (and presumably replication in the CNS) is necessary to induce impairments so the concern raised here may not be applicable to some vectors."*
• This is not so much a "claim", but an important suggestion to the field that additional studies of behavioral morbidity be included in preclinical safety trials.

10. "*Line 483, 671: it is not necessary to thank Dr. Knipe as he is an author on the study*."
• The acknowledgement has been deleted.

11. *Line 495: is this the correct JAX# for these mice?*
• We have confirmed that Strain #034848-JAX is correct for 5X FAD mice.

Again, we wish to thank the reviewers for excellent comments that have resulted in a significantly strengthened manuscript. We look forward to receiving the reviews/decision on the revised manuscript.

On behalf of all authors,

Regards,

David A. Leib, PhD.
Professor and Chair
Geisel School of Medicine at Dartmouth
Department of Microbiology and Immunology
Lebanon, NH 03756.

Tel: 603-646-5232
david.a.leib@dartmouth.edu
http://dms.dartmouth.edu/leib/
